# Differential Associations of IL-4 With Hippocampal Subfields in Mild Cognitive Impairment and Alzheimer’s Disease

**DOI:** 10.3389/fnagi.2018.00439

**Published:** 2019-01-17

**Authors:** Virginia Boccardi, Eric Westman, Luca Pelini, Olof Lindberg, J-Sebastian Muehlboeck, Andrew Simmons, Roberto Tarducci, Piero Floridi, Pietro Chiarini, Hilkka Soininen, Iwona Kloszewska, Magda Tsolaki, Bruno Vellas, Christian Spenger, Lars-Olof Wahlund, Simon Lovestone, Patrizia Mecocci

**Affiliations:** ^1^Department of Medicine, Institute of Gerontology and Geriatrics, University of Perugia, Perugia, Italy; ^2^Division of Clinical Geriatrics, Department of Neurobiology, Care Sciences and Society, Karolinska Institutet, Stockholm, Sweden; ^3^Department of Neuroimaging, Institute of Psychiatry, Psychology & Neuroscience, King’s College London, London, United Kingdom; ^4^Division of Medical Physics, Perugia University Hospital, Perugia, Italy; ^5^Division of Neuroradiology, Perugia University Hospital, Perugia, Italy; ^6^Department of Neurology, University of Eastern Finland – Kuopio University Hospital, Kuopio, Finland; ^7^Department of Old Age Psychiatry and Psychotic Disorders, Medical University of Łódź, Łódź, Poland; ^8^3rd Department of Neurology, Aristotle University of Thessaloniki, Thessaloniki, Greece; ^9^University of Toulouse, INSERM 1027, Gérontopôle, Toulouse, France; ^10^Department of Clinical Science, Intervention and Technology, Karolinska Institutet, Stockholm, Sweden; ^11^Department of Psychiatry, Warneford Hospital, University of Oxford, Oxford, United Kingdom

**Keywords:** aging, Alzheimer’s disease, mild cognitive impairment, inflammation, inflammatory markers

## Abstract

**Background/Aims:** We aimed to assess the association between in volumetric measures of hippocampal sub-regions – in healthy older controls (HC), subjects with mild cognitive impairment (MCI) and AD- with circulating levels of IL-4.

**Methods:** From AddNeuroMed Project 113 HC, 101 stable MCI (sMCI), 22 converter MCI (cMCI) and 119 AD were included. Hippocampal subfield volumes were analyzed using Freesurfer 6.0.0 on high-resolution sagittal 3D-T1W MP-RAGE acquisitions. Plasmatic IL-4 was measured using ELISA assay.

**Results:** IL-4 was found to be (a) positively associate with left subiculum volume (β = 0.226, *p* = 0.037) in sMCI and (b) negatively associate with left subiculum volume (β = -0.253, *p* = 0.011) and left presubiculum volume (β = -0.257, *p* = 0.011) in AD.

**Conclusion:** Our results indicate a potential neuroprotective effect of IL-4 on the areas of the hippocampus more vulnerable to aging and neurodegeneration.

## Introduction

A large body of evidence supports an extensive bi-directional communication between the immune system and the central nervous system (Barrientos et al., 2015), with peripheral immune activation able to modulate behavioral and cognitive aspects as well as neuroplasticity (Konsman et al., 2001; Yirmiya and Goshen, 2011; Picco et al., 2014; Marsland et al., 2015). Because of this communication, neural activity is altered quite dramatically during and following a peripheral immune challenge (Maier, 2003; Maher et al., 2005), leading to *de novo* cytokine production within the brain parenchyma. This response is driven primarily by activated microglial cells (Turrin et al., 2001) that, in response to injury, acquire a reactive inflammatory phenotype, also referred to as the “M1 or classic phenotype." The classic inflammatory phenotype is characterized by increased microglia proliferation, morphological changes, and release of several molecules including cytokines, chemokines, reactive oxygen species, and nitric oxide (Kettenmann et al., 2011). In contrast to the classic inflammatory phenotype, microglia can acquire what has been termed the “alternative neuroprotective or M2 phenotype” that plays a central role in regenerative processes (Colton, 2009). Most of the research on this topic has focused on the immune-mediated detrimental effects of inflammation on neural plasticity, specifically by reducing hippocampal long-term potentiation (LTP) induction and maintenance (Goshen and Yirmiya, 2007). The notion that microglia have differential effects on neurogenesis, depending on the expressed immune phenotype, is supported by Butovsky et al. (2006), who demonstrated how neural progenitor cells cultured with IL-4-stimulated microglia showed an increased proportion of new neurons and oligodendrocytes. Additionally, stimulating microglial cells with IL-4 increased microglia expression of IGF-1 that is known to support neurogenesis (Annenkov, 2009). Although the effects of IL-4, an anti-inflammatory cytokine produced by Th2 cells, in aging and Alzheimer’s disease (AD) have been reported in few publications, the role of this cytokine in counteracting age-related inflammatory changes in the brain is supported by the consistency of results across studies. Maher et al. (2005) found that an increase in IL-1β and IL-6 observed in the hippocampus of aged animals is accompanied by a decrease in hippocampal IL-4.

Moreover, IL-4 has been shown to induce clearance of oligomeric Aβ peptides and attenuate *in vitro* and *in vivo* Aβ-related neuroinflammation. In fact, IL-4 induces the clearance of oligomeric Aβ by primary rat microglial cells via increased expression of scavenger receptor CD36 and the Aβ-degrading enzymes neprilysin (NEP) (Shimizu et al., 2008). Furthermore, *in vivo* injection of IL-4/IL-13 mixture led to a reduction in brain Aβ levels and to an improvement in cognitive function in amyloid precursor protein (APP23) transgenic mice (Kawahara et al., 2012). IL-4-based treatment and immunization have been reported to reduce Aβ production and elicit a neuroprotective effect in APP+PS1 transgenic mice (Kiyota et al., 2010).

Consistent evidence supports a significant vulnerability of the hippocampus to aging and to some different neurological and psychiatric conditions. The most common finding is an age-related volume reduction (Small et al., 2011). However, the magnitude of age differences in hippocampal volume and the rate of hippocampal shrinkage vary widely across studies (Raz, 2009), and the mechanisms underlying age-related changes in the hippocampus remain unclear. Volumetric shrinkage of the hippocampus is also regarded as an indicator of disease-related neuronal injury in AD and, also, strongly associated with Aβ deposition, increased functional activation and clinical progression in subjects with Mild Cognitive Impairment (MCI) (Grundman et al., 2002; Huijbers et al., 2015). The hippocampus is composed of several histologically defined and interconnected subfields including parasubiculum, presubiculum, subiculum, Cornu Ammonis (1, 3, and 4). Until recently, *in vivo* studies of hippocampal aging and AD did not take into account its cytoarchitectonic and functional heterogeneity. Using the volume of the whole hippocampus may obscure the differential sensitivities of its components to specific risk or protective factors. For example, one of the hippocampal subfields, CA1, is particularly sensitive to hypoxia, ischemia, and hyperglycemia while CA3 and DG are usually spared (De Flores et al., 2015a,b). In *in vivo* MRI studies, subiculum has been consistently found to be the most vulnerable substructure to age and neurodegeneration in the hippocampus (Frisoni et al., 2008). This vulnerability is supported by the developmental theory, according to which the last cerebral structures to develop, such as subiculum and presubiculum, are the least resistant to the effects of several stressors (Grieve et al., 2005; Kalpouzos et al., 2009; La Joie et al., 2010). To the best of our knowledge, circulating IL-4 levels have not been investigated in association with the neuroanatomic profile of the hippocampal subfields in normal aging and AD to date. In this perspective, we aim to evaluate group wise volumetric differences in the substructures of the hippocampus and to investigate the existence of a relationship between IL-4 and hippocampal subfields in the AddNeuroMed cohort, a large European imaging dataset from healthy and cognitively impaired old age subjects.

## Materials and Methods

### Subjects

Three hundred and fifty-three subjects from the AddNeuroMed study were included (113 HC, 121 MCI, 119 AD). The AddNeuroMed study is an integrated project funded by the European Union Sixth Framework Program aimed at establishing and validating novel biomarkers of disease and treatment based upon *in vitro* and *in vivo* human and animal models of AD. Data were collected from six participating sites across Europe: University of Kuopio, Finland; University of Perugia, Italy; Aristotle University of Thessaloniki, Greece; King’s College London, United Kingdom; University of Łódź, Poland; and University of Toulouse, France (Lovestone et al., 2009).

Subjects were patients who attended local memory clinics and received a diagnosis of MCI and early AD, while HC subjects were recruited from non-related members of the patient’s families, caregiver relatives, and social centers for the elderly or General Practitioner (GP) surgeries. Informed and written consent was obtained from all subjects, and the ethical review boards of each participating country approved the study. The general inclusion and exclusion criteria were as follows:

#### AD

Diagnosis established according to the National Institute of Neurological and Communicative Disorders and Stroke and the Alzheimer’s disease and Related Disorders Association (NINCDS-ADRDA), Diagnostic, and Statistical Manual of Mental Disorders IV (DSM IV) criteria. Subjects were excluded from the study if any psychiatric or neurological illness other than AD was present, and if subjects presented with a systemic illness or signs of organ failure.

#### MCI

Subjects with an MMSE score between 24 and 30, subjective memory complaint with preserved activities of daily living, Clinical Dementia Rating (CDR) score of 0.5, Geriatric Depression Scale (GDS) score less than or equal to 5, absence of dementia by NINCDS-ADRDA criteria (Frisoni et al., 2011). A 12 month follow up was used to determine whether MCI subjects converted to AD (MCI converters, cMCI) or remained clinically stable (MCI stable, sMCI).

#### HC

MMSE scores between 24 and 30, CDR score of 0, no presence of neurological or psychiatric illness, and non-demented.

### MRI Acquisition

Standardized MRI data acquisition techniques were in place for AddNeuroMed to ensure homogeneity across data acquisition sites, using the ADNI protocol ^1^. The imaging protocol included a 1.5T high-resolution T1 weighted sagittal 3D MP-RAGE volumes (voxel size 1.1 × 1.1 × 1.2 mm^3^), and axial proton density with T2 weighted fast spin echo images. A comprehensive quality control procedure was carried out on all MR images according to the AddNeuroMed quality control framework (Simmons et al., 2009).

### Hippocampal Subfields Segmentation

Image analysis was carried out using the Freesurfer image analysis pipeline (version 6.0.0). These procedures have previously been described (Fischl et al., 2002, 2004; Ségonne et al., 2004). Initially, volumetric segmentation involved the removal of non-brain tissue using a hybrid watershed/surface deformation procedure (Ségonne et al., 2004), automated Talairach transformation, segmentation of the subcortical white matter and deep gray matter volumetric structures (Fischl et al., 2004). Automated segmentation of the hippocampus was performed to define anatomical subfield labels using a Bayesian modeling approach and a computational model of the areas surrounding the hippocampus. An atlas mesh had previously been built and validated from manual delineations in ultra-high resolution MRI scans of 10 individuals (Van Leemput et al., 2009; Westman et al., 2013). These delineations include the parasubiculum, presubiculum, subiculum, CA1 (Cornu Ammonis), CA3 (Cornu Ammonis), CA4 (Cornu Ammonis), hippocampal tail, molecular layer HP (hippocampus), GC-ML-DG (Granule cell Layer of Dentatus Gyrus), fimbria, HATA (hippocampus amygdala-transition-area) and fissure. All subfield measures were normalized by the subject’s intracranial volume (ICV) derived from Freesurfer using the following formula: volume norm = volume raw × 1,000/ICV in mm^3^) (Voevodskaya et al., 2014). Data were processed using the Hive database system (La Joie et al., 2013).

### Sample Preparation and Data Acquisition

All participants were required to fast for 2 h before blood sample collection; only water or fluids containing no milk or sugar were allowed during the fasting period. Plasma samples were collected using EDTA coated tubes and centrifuged at 3000 rpm for 8 min at 4°C before being aliquoted and then frozen at -80°C. Plasma samples were analyzed with ELISA (Bio-Rad Laboratories, Hercules, CA, United States) for the assessment of peripheral IL-4. The samples were prepared according to the manufacturer’s instructions. All samples and standards were run in duplicate and were measured as pg/ml. The system running protocol was set according to the manufacturer’s guidelines.

### Statistical Analysis

The observed data are presented as means ± SD. To approximate a normal distribution, plasma IL-4 levels were logarithmically transformed for all calculations. Chi-square test, One-way ANOVA followed by Bonferroni *post hoc* analysis and correlation by *r* Pearson were performed where appropriated. Linear regression analysis was conducted using hippocampal subfield volumes as dependent variables and log-transformed IL-4 measures along with age, education, APOE4 status and MMSE score as independent variables. All *p*-values presented are 2-tailed, and *p* ≤ 0.05 was chosen for levels of significance. Statistical analysis was conducted using SPSS Statistics (Version 21.0, SPSS Inc., United States).

## Results

### Demographics

Three hundred and fifty-three subjects were included from the AddNeuroMed study: 113 HC, 101 sMCI, 20 cMCI and 119 AD. Demographic, genetic and neuropsychological data are shown in Table 1. Significant differences across groups were found in years of education (*p* < 0.001), MMSE score (*p* < 0.001) and APOE4 genotype (*p* < 0.001), while no gender difference was found among groups. AD was significantly less educated, with lower scores at baseline in MMSE and statistically significant higher prevalence of APOE4 allelic status as compared with HC subjects.

**Table 1:** Demographic, clinical and neuropsychological data by diagnosis.

	HC	sMCI	cMCI	AD
	(n 113)	(n 101)	(n 20)	(n 119)	*p*
Gender (n,M/F)	52/61	53/48	12/8	48/71	0.094
Age (years)	73.0 ± 6.5	74.7 ± 5.4	72.2+6.5	75.6 ± 6.0^a^	0.005
Years of education	10.7 ± 4.7	8.7 ± 4.2	9.7+4.4	7.9 ± 3.9^b^	<0.0001
MMSE score	29.0 ± 1.2	27.1 ± 1.6	26.9+1.9	20.7 ± 4.6^b^	<0.0001
APOE4 genotype (+ive, -ive)	32/74	25/62	12/5	58/44^b^	<0.0001


*HC, Healthy controls; sMCI, stable MCI; cMCI, converter MCI; AD, Alzheimer’s disease; +ive, positive; -ive, negative where available. Data are shown as mean ± standard deviation. Chi-square was used for gender and APOE4 genotype comparisons. ANOVA was used for age, education, and MMSE. Bonferroni pairwise comparisons: ^a^AD vs. HC p = 0.010; ^b^AD vs. HC p < 0.0001.*

### Hippocampal Subfields and Clinical Cognitive Diagnosis

As expected, total left hippocampal volume significantly differed among groups (3059.16 ± 324.51 in HC, 2820.47 ± 435.44 in sMCI, 2628.16 ± 441.63 in cMCI and 2338.08 ± 411.45 in AD; *p* < 0.0001). Then, hippocampal subfields were measured in all groups to detect the regional distribution of hippocampal atrophy in relation to clinical diagnosis. As shown in Table 2, comparisons of left volumes of parasubiculum, presubiculum, subiculum, CA1, CA3, CA4, hippocampal tail molecular layer HP and GC-ML-DG fimbria, HATA, fissure reported statistically significant differences across all diagnostic groups even after correction for multiple covariates including age, gender, education and APOE4 allelic status, with the exception of parasubiculum (*p* = 0.130). Total right hippocampal volume significantly differed among groups (3157.78 ± 353.90 in HC, 2929.80 ± 453.25 in sMCI, 2690.81 ± 541.43 in cMCI and 2478.33 ± 454.29 in AD). Right hippocampus volumes were significantly reduced in all hippocampal areas even after correction for multiple covariates.

**Table 2:** Hippocampal subfield differences in HC, sMCI, cMCI and AD subjects.

	HC	sMCI	cMCI>	AD	*p*
**Left hippocampus**					
Parasubiculum	54.53 ± 12.84	53.26 ± 14.65	52.85 ± 9.15	48.03 ± 14.05	0.002
Presubiculum	275.48 ± 40.85^b,c^	254.62 ± 43.00^a^	239.84 ± 46.39^a^	211.67 ± 45.87^a,b^	<0.0001
Subiculum	392.66 ± 49.83^b,c^	355.95 ± 59.89^a^	320.31 ± 65.63^a^	287.32 ± 63.69^a,b^	<0.0001
CA1	576.83 ± 66.54^b,c^	533.88 ± 92.98^a^	496.76 ± 74.55^a^	453.15 ± 84.91^a,b^	<0.0001
CA3	185.31 ± 25.82^c^	172.38 ± 36.76	160.60 ± 36.39	140.17 ± 29.81^a,b^	<0.0001
CA4	225.07 ± 26.28^b,c^	210.26 ± 35.43^a^	194.86 ± 36.44^a^	173.77 ± 32.74^a,b^	<0.0001
Hippocampal tail	482.53 ± 82.53^b,c^	447.94 ± 47.94^a^	434.83 ± 34.83^d^	375.40 ± 75.40^a,b,c^	<0.0001
Molecular Layer	502.23 ± 57.01^b,c^	460.04 ± 75.57^a^	420.54 ± 75.14^a^	377.18 ± 72.62^a,b^	<0.0001
GC-ML-DG	258.38 ± 32.00^b,c^	239.05 ± 41.86^a^	221.21 ± 43.09^a^	197.55 ± 37.54^a,b^	<0.0001
Fimbria	53.11 ± 19.59	46.04 ± 20.96	41.27 ± 23.00	33.91 ± 16.96^a,b^	<0.0001
HATA	52.98 ± 10.47^b^	46.99 ± 10.91^a^	45.10 ± 11.57	39.88 ± 10.19^a,b^	<0.0001
Fissure	173.24 ± 33.69	172.90 ± 35.50	164.53 ± 38.00	155.70 ± 40.37	0.001
**Right hippocampus**					
Parasubiculum	54.36 ± 10.85	48.82 ± 12.17	44.79 ± 11.65	45.57 ± 14.33^a^	<0.0001
Presubiculum	264.87 ± 41.88^b,c^	238.53 ± 42.71^a^	217.15 ± 50.25^a^	201.78 ± 44.97^a,b^	<0.0001
Subiculum	396.67 ± 54.08^b,c^	361.05 ± 62.95^a^	319.79 ± 71.62^a^	294.43 ± 68.13^a,b^	<0.0001
CA1	594.36 ± 69.61^c,^	561.17 ± 93.71	518.50 ± 94.66	484.82 ± 98.42^a,b^	<0.0001
CA3	203.08 ± 29.57	190.42 ± 37.92	177.52 ± 45.46	159.81 ± 32.88^a,b^	<0. 0001
CA4	239.61 ± 29.17^c^	226.84 ± 35.75	211.40 ± 44.57^a^	192.53 ± 35.08^a,b^	<0.0001
Hippocampal tail	511.44 ± 72.91^b^	477.38 ± 73.86^a^	457.96 ± 88.35	409.96 ± 69.14^a,b^	<0.0001
Molecular Layer	516.25 ± 60.60^b,c^	477.02 ± 78.79a	434.41 ± 94.13a	396.58 ± 80.05^a,b^	<0.0001
GC-ML-DG	273.52 ± 33.90^b^	257.45 ± 42.57^a^	236.92 ± 49.14^a^	217.72 ± 41.24^a,b^	<0.0001
Fimbria	49.59 ± 19.04^b,c^	41.08 ± 17.78^a^	30.43 ± 16.43^a^	31.88 ± 18.05^a^	<0.0001
HATA	53.99 ± 10.56^c^	50.00 ± 11.16^c^	41.89 ± 11.21^a^	43.20 ± 11.69^a,b^	<0.0001
Fissure	187.91 ± 35.45	190.76 ± 36.78	180.60 ± 41.90	170.02 ± 39.06^b^	<0.0001


*Hippocampal subfields volumes are presented as absolute measures (mm^3^). HC, Healthy controls; sMCI, stable MCI; cMCI, converter MCI; AD, Alzheimer’s disease; CA1, Cornu ammonis 1; CA2, Cornu ammonis 2; CA3, Cornu ammonis 3; CA4, Cornu ammonis 4; HATA, hippocampus amygdala-transition-area; GC-ML-DG, Granule cell Molecular Layer of Dentatus Gyrus. Normalized hippocampal volumes were used in ANOVA with Bonferroni pairwise comparisons. ^a^ = p < 0.005 vs. HC; ^b^ = p < 0.005 vs. sMCI; ^c^ = p < 0.005 vs. cMCI.*

### Relationship Between IL-4 and Hippocampal Subfields in the Overall Sample and Each Diagnostic Group

In all population, a trend in a reduction for plasmatic IL-4 levels along with age (*r* = -0.106, *p* = 0.073) was found. No difference in IL-4 levels (*p* = 0.609) and ApoE genotypes (*p* = 0.468) was found between gender. Stratifying the sample population by clinical diagnosis, no difference in IL-4 levels was found among groups (*p* = 0.873). At multivariate linear regression, plasma IL-4 levels were positively associated with left subiculum volume (β = 0.226, *p* = 0.037) in sMCI; contrarily, IL-4 levels were negatively associated with left subiculum volume (β = -0.253, *p* = 0.011) and left presubiculum volume (β = -0.257, *p* = 0.011) in AD. No significant associations were found in cMCI and HC.

## Discussion

Collectively our data show a significant volume loss in hippocampal subfields of subjects with AD and a positive association between plasmatic IL-4 and volumetric measures of left subiculum in sMCI subjects while a negative association with left subiculum and left presubiculum in AD.

According to previous evidence using data from both the AddNeuroMed and the ADNI project, as compared with healthy subjects, MCI and AD presented a bilateral hippocampal atrophy, involving all subregions (Boutet et al., 2014; De Flores et al., 2015c). We found that parasubiculum, HATA, and fimbria volumes were the most stable subfield measure across groups. Hippocampal tail, molecular layer, subiculum, and CA1 showed the most severe involvement and more pronounced in the left-brain. Our findings support the hypothesis that the progression of volume loss is heterogeneous across the various hippocampal subfields, with distal areas being the most significant and early affected by the ongoing neurodegenerative process from a cognitively healthy status to AD. This is consistent with results from a neuropathologic investigation where disproportionate atrophy of the subicular complex with respect to CA fields (CA1, 2, 3, and 4) and the dentate gyrus was described (Mizutani and Kasahara, 1997). The particularly severe volume loss of these structures was attributed to fibrillary gliosis in the context of an early and severe involution of the perforant pathway penetrating the hippocampus through the subicular complex, in its course from the entorhinal cortex to the dentate gyrus. Another histological study reported major neuronal losses in AD patients in CA1 (68%) as well as in the subiculum (47%) in comparison with age-matched controls (West et al., 1994). In neuroimaging studies direct volumetry in AD patients reported significant atrophy in all, or almost all, investigated subfields, the major atrophy being located in CA1 (Mueller et al., 2010; La Joie et al., 2013; Boutet et al., 2014; Voevodskaya et al., 2014). Carlesimo et al. (2015), using an automated hippocampal segmentation protocol in a smaller cross-sectional imaging dataset, reported the early and severe involvement of the subiculum and presubiculum in patients with MCI and AD compared to HC. According to our findings, other studies have also shown that CA1 and subiculum atrophy was present at a very early stage of AD, as it was detectable in cognitively healthy individuals that later developed MCI or AD (Csernansky et al., 2005). Altogether, our report stresses the importance of hippocampal subfield measurements as sensitive markers of AD processes and emphasizes their potential role as biomarkers for early AD detection.

IL-4 is a cytokine that functions as a potent regulator of immunity, secreted primarily by mast cells, Th2 cells, eosinophils, and basophils. Initially identified by Howard and Paul (Howard et al., 1982) as a co-mitogen of B-cells, IL-4 was subsequently shown to be an important player in leukocyte survival under both physiological and pathological conditions such as Th2 cell-mediated immunity (Hsieh et al., 1992; Seder et al., 1992), IgE class switching in B cells (Geha et al., 2003), and tissue repair and homeostasis through “alternative” macrophage activation, M2 (Gordon, 2003). The M2 group of stimuli arose from the initial IL-4 observations, and they are grouped mainly due to their ability to antagonize prototypic inflammatory responses and markers. IL-4 has shown beneficial effects in the aging hippocampus of rodents, in particular promoting neuroplasticity and LTP, a cellular mechanism of memory, maintenance and rescue, and in counteracting age-related pro-inflammatory changes. Indeed, while IL-1β significantly contributes to the impairment of LTP along with age (Bellinger et al., 1993; Lynch, 2010) work by Maher et al. (2005) revealed that the age-related decrease in hippocampal IL-4 concentration, coupled with the downregulation of IL-4-associated signaling, also contributes to LTP impairment. In murine models of AD introduction of an adeno-associated viral vector encoding IL-4 into the hippocampus resulted in sustained expression of IL-4, reduced astro/microgliosis, reduced Aβ oligomerization and deposition, and enhanced neurogenesis. Shimizu et al. (2008) found that IL-4 treatment of rat primary-type 2 microglia enhanced uptake and degradation of oligomeric Aβ 1–42 via the induction of the expression of the scavenger receptor CD36 and the Aβ degrading enzymes neprilysin (NEP). In this study, we aimed to test the existence of a relationship between plasma IL-4 levels and volumetric measures of hippocampal subfields. In agreement with previous works (Swardfager et al., 2010) concentrations of IL-4 did not differ across diagnostic groups or APOE 𝜀4 presence. Using multiple linear regression models, we found a significant and positive association between circulating IL-4 levels and left subiculum in sMCI after controlling for multiple covariates. On the contrary, a negative and significant association was found among IL-4 levels and left subiculum and left presubiculum in subjects affected by AD. On the basis of previous findings that indicated an involvement of IL-4 in the pathogenesis of AD (Sachin et al., 2012) and the BBB permeability to most plasmatic inflammatory mediators (Lyons et al., 2007) these results suggest a possible effect of circulating IL-4 levels in counteracting neurodegenerative changes within the most vulnerable areas of the hippocampus in the prodromal stages of AD. Butovsky et al. (2006) reported that microglia activated by IL-4 induced both neurogenesis and oligodendrogenesis, with an insulin-like growth factor-I (IGF-I) mediated effect. Further analysis by the same authors showed that IL-4 produces additional neurogenic factors besides IGF-I. A protective effect toward neurodegeneration in the AD pathogenesis might explain the persistence of a positive relationship with volumetric measures in subfields from the subicular complex among subjects in the initial stage of the disease. Accordingly, cMCI showed lower IL-4 plasmatic levels as compared with sMCI and probably more susceptible to conversion. Longitudinal and larger studies are needed to confirm such a hypothesis. Our results suggest a tight link between circulating inflammation markers and structural brain measures (Frodl and Amico, 2014; Zhang et al., 2016) pointing out that, despite IL-4 does not show any detectable change in plasmatic levels when individuals with different cognitive status are compared, it may be independently associated with structural integrity of the most vulnerable areas of the hippocampus especially at early stage of disease. The relatively large sample size and the evaluation with a comprehensive MRI protocol, are the main strengths. This study has some limitations. Segmentation of small structures on standard resolution T1-weighted MR appears particularly challenging as the limited spatial resolution of the images may compromise the accurate delineation of subfield boundaries, and especially given the rather large number (7) of hippocampal substructures segmented by FreeSurfer. The Bayesian inferential method deployed on FreeSurfer 6.0 has not been robustly validated against a gold- standard method, such as manual segmentation (Wolf et al., 2015). However, automatic segmentation using FreeSufer still provides highly useful information of the hippocampus as the *ex vivo* atlas significantly outperforms the *in vivo* atlas in the segmentation of the whole hippocampus. Moreover, by using an automated segmentation, it allows for a more reproducible and automatic fashion of segmenting the hippocampus into its subregions instead of the typical labor intensive and biased method of manual hippocampus segmentation. Although we adjusted for major potential confounders, residual confounding (i.e., vascular diseases such as hypertension and diabetes, medication use such as NSADs, smoking have been described to influence ILs levels) is possible given the observational design of the study. IL-4 was only evaluated once in our study. A within-group design facilitates an examination of intra-individual variability in cytokine levels and profiles over time. Indeed, a longitudinal design would have been more adequate to our purpose, allowing us to investigate on the relationship that IL-4 might have with the rate of hippocampal subfields volume loss among subjects in the prodromal stages of AD.

## Conclusion

In summary, results from our study indicate a widespread distribution of volume loss in the hippocampus of AD patients, with substantial involvement of the hippocampal tail, CA1, subiculum and molecular layer. Secondly, this is the first *in vivo* report of peripheral IL-4 relationships with MRI-derived structural markers of neuronal integrity. Our data embrace the hypothesis previously raised by studies on animal models of aging and AD of a neuroprotective effect of IL-4 toward hippocampus, bringing in evidence of considerable relationships with its structural properties, and particularly involving the distal hippocampal areas.

## Author Contributions

VB, LP, and PM planned the project, analyzed the data, interpreted the results, and wrote the manuscript. EW and OL generated all the data. J-SM, AS, RT, PF, PC, HS, IK, MT, BV, CS, L-OW, and SL interpreted and discussed the data.

## Conflict of Interest Statement

The authors declare that the research was conducted in the absence of any commercial or financial relationships that could be construed as a potential conflict of interest.

## Footnotes

1 http://adni.loni.usc.edu/methods/mri-tool/mri-analysis/
